# Enhanced biglycan gene expression in the adipose tissues of obese women and its association with obesity-related genes and metabolic parameters

**DOI:** 10.1038/srep30609

**Published:** 2016-07-28

**Authors:** Jimin Kim, Seul Ki Lee, Ji-min Shin, Un-woo Jeoun, Yeon Jin Jang, Hye Soon Park, Jong-Hyeok Kim, Gyung-Yub Gong, Taik Jong Lee, Joon Pio Hong, Yeon Ji Lee, Yoon-Suk Heo

**Affiliations:** 1 Department of Physiology, Cell Dysfunction Research Center, University of Ulsan College of Medicine, Seoul, Korea; 2 Department of Family Medicine, University of Ulsan College of Medicine, Seoul, Korea; 3 Department of Obstetrics and Gynecology, University of Ulsan College of Medicine, Seoul, Korea; 4 Department of Pathology, University of Ulsan College of Medicine, Seoul, Korea; 5 Department of Plastic Surgery, University of Ulsan College of Medicine, Seoul, Korea; 6 Department of Family Medicine, Inha University, College of Medicine, Incheon, Korea; 7 Department of General Surgery, Inha University, College of Medicine, Incheon, Korea

## Abstract

Extracellular matrix (ECM) remodeling dynamically occurs to accommodate adipose tissue expansion during obesity. One non-fibrillar component of ECM, biglycan, is released from the matrix in response to tissue stress; the soluble form of biglycan binds to toll-like receptor 2/4 on macrophages, causing proinflammatory cytokine secretion. To investigate the pattern and regulatory properties of biglycan expression in human adipose tissues in the context of obesity and its related diseases, we recruited 21 non-diabetic obese women, 11 type 2 diabetic obese women, and 59 normal-weight women. Regardless of the presence of diabetes, obese patients had significantly higher biglycan mRNA in both visceral and subcutaneous adipose tissue. Biglycan mRNA was noticeably higher in non-adipocytes than adipocytes and significantly decreased during adipogenesis. Adipose tissue biglycan mRNA positively correlated with adiposity indices and insulin resistance parameters; however, this relationship disappeared after adjusting for BMI. In both fat depots, biglycan mRNA strongly correlated with the expression of genes related to inflammation and endoplasmic reticulum stress. In addition, culture of human preadipocytes and differentiated adipocytes under conditions mimicking the local microenvironments of obese adipose tissues significantly increased biglycan mRNA expression. Our data indicate that biglycan gene expression is increased in obese adipose tissues by altered local conditions.

In addition to its role in fat storage, adipose tissue is a secretory organ that releases several biologically active molecules. Adipose tissue is also believed to play a major role in the pathogenesis of metabolic and inflammatory obesity-related disorders^1^,^2^. During the development of obesity, excess metabolic energy induces several changes in adipose tissues, including adipocyte hypertrophy and hyperplasia, inflammatory cell accumulation, and neovascularization. To accommodate these changes, extracellular matrix (ECM) remodeling occurs in which the existing ECM is degraded and new ECM is produced^3^. Notably, transcriptomic signature analysis of adipose tissue from obese humans revealed a significant upregulation of ECM component genes and a strong interrelationship with inflammatory processes^4^. Accordingly, the identification of ECM components that are related to adipose inflammation and metabolic derangements in obesity is of considerable interest.

Biglycan is a ubiquitously expressed ECM component and class I member of the small leucine-rich proteoglycan family^5^. It consists of a 42-kDa protein core with two N-terminal chondroitin/dermatan sulfate side chains^6^. It is believed to organize matrix assembly by interacting with ECM components such as collagens and elastins^7^,^8^,^9^ and is normally sequestered under physiological conditions. However, in response to tissue stress or damage, the soluble form of biglycan is produced either via partial ECM proteolysis or via *de novo* synthesis by activated macrophages and other resident cells^10^,^11^. Unsequestered biglycan can bind to macrophage toll-like receptors (TLRs) 2 and 4, thereby inducing secretion of proinflammatory cytokines such as tumor necrosis factor α (TNF-α)^12^,^13^,^14^,^15^ and IL-1β^12^. Thus, besides providing structural support to adipocytes and other cells, biglycan may also regulate adipose tissue function.

In *Psammomys obesus*, biglycan expression was strikingly higher in adipose tissues than in all other tissues examined^16^. In addition, it was markedly upregulated in the adipose tissues of obese and diabetic individuals compared with lean, normal glucose-tolerant individuals^16^. In rodents, a high-fat diet significantly increases adipose tissue biglycan mRNA^17^,^18^ and protein^19^ expression. In addition, in contrast to wild-type mice, high-fat diet-induced glucose intolerance was ameliorated in biglycan knockout mice, implicating a role for biglycan in glucose metabolism^17^. However, studies on biglycan expression in human adipose tissues are limited^20^.

The present study was undertaken to determine the pattern and regulatory properties of biglycan expression in human adipose tissues in the context of obesity and its related diseases. We compared biglycan expression levels in two separate fat depots, abdominal subcutaneous adipose tissue (SAT) and visceral adipose tissue (VAT), in obese women (with or without type 2 diabetes) and normal-weight women. We investigated the association of biglycan mRNA expression in adipose tissues with other metabolic parameters and the abdominal fat distribution. Furthermore, to understand how biglycan expression is induced upon obesity, we incubated human preadipocytes as well as differentiated adipocytes in a series of culture environments that could mimic the conditions during obesity.

## Results

### Characteristics of the study subjects

The clinical characteristics of the normal-weight control and non-diabetic or type 2 diabetic obese groups are described in Supplementary Table 1. Their metabolic characteristics are summarized in Table 1. The non-diabetic obese patients were significantly younger, but their body mass index (BMI) was significantly higher than either the normal-weight control or obesity with diabetes groups. Compared with normal-weight control patients, obese patients with or without diabetes displayed significantly higher blood pressure (BP), homeostasis model assessment of insulin resistance (HOMA-IR), adipocyte size, and circulating concentrations of insulin, LDL-cholesterol, triglyceride, high sensitivity C-reactive protein (hs-CRP), and leptin. Abdominal computerized tomography (CT) also showed significantly greater total abdominal adipose tissue (TAT), VAT, and SAT areas in obese patients than the control group. However, an index of visceral obesity, the VAT:SAT ratio (VSR), was significantly elevated only in the diabetic patients and not in non-diabetic obese patients.

### Elevated biglycan mRNA expression in adipose tissues of obese patients

**Figure 1:**
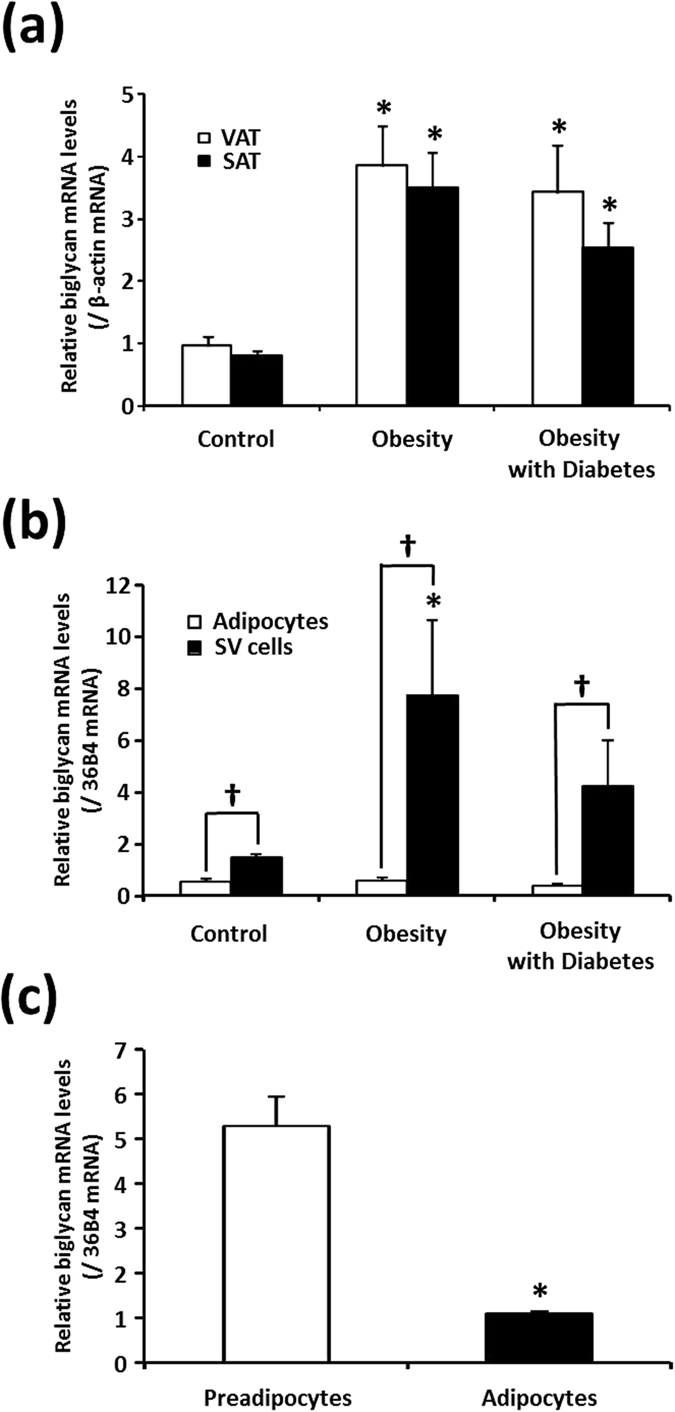
Biglycan mRNA expression in human adipose tissues. (**a**) Biglycan mRNA in the visceral (VAT) and subcutaneous (SAT) adipose tissue of normal-weight control (*n* = 59), non-diabetic obesity (*n* = 21), and obesity with type 2 diabetes (*n* = 11) groups. Biglycan mRNA levels are normalized to β-actin gene expression. Data were log-transformed before statistical analyses and are shown here as the mean ± s.e.m. on the original (back-transformed) scale (^*^P < 0.05 vs control for the corresponding fat depot). Data were analyzed by ANOVA with Tukey’s *post hoc* test. (**b**) Biglycan mRNA levels in adipocyte and stromal vascular (SV) cell fractions obtained from adipose tissues. VAT of normal-weight women (*n* = 9) who underwent benign gynecological surgery and non-diabetic obese women (*n* = 6) and obese women with diabetes (*n* = 5) who underwent laparoscopic Roux-en-Y gastric bypass surgery was removed during surgery and digested with collagenase to separate floating adipocytes and non-floating SV cell fractions. Biglycan mRNA levels are normalized to 36B4 gene expression (^†^*P* < 0.05 by paired *t*-test; ^*^P < 0.05 vs control for the corresponding cell fraction by ANOVA with Tukey’s *post hoc* test). (**c**) Biglycan mRNA in human preadipocytes before and after differentiation to adipocytes. Preadipocytes were isolated from human SAT and differentiated to adipocytes as described in the METHOD section. Biglycan mRNA levels are normalized to 36B4 gene expression (*n* = 3; ^*^P < 0.05 vs preadipocytes by paired *t*-test).

Within the three groups, the biglycan mRNA levels in VAT and SAT were not significantly different (*P* = 0.20 or higher; Fig. 1a). Within individuals, the VAT and SAT biglycan mRNA levels were strongly correlated (*r* = 0.493; *P* < 0.001; *n* = 91, data not shown). Thus, biglycan mRNA was expressed at a similar level in the two fat depots. On the other hand, in obese patients, regardless of the presence of type 2 diabetes, both VAT and SAT biglycan mRNA levels were significantly higher (3–4-fold) than in normal-weight control subjects (Fig. 1a).

Next, we separated the adipocyte and stromovascular (SV) cell fractions from the VAT samples of the three groups (Fig. 1b). In all three groups, we found that the biglycan mRNA level was significantly higher in the SV cell fraction than in the adipocyte fraction. In addition, compared with the normal-weight control patients, obese patients had higher biglycan mRNA levels in the SV cell fractions, although the differences were only statistically significant in non-diabetic obese patients and not in diabetic patients. In adipocyte fractions, the biglycan mRNA levels were not significantly different among the three groups. Consistently with this, when we measured biglycan mRNA in preadipocytes isolated from human adipose tissues before and after differentiation, the expression level was about five times higher in preadipocytes than in fully differentiated adipocytes (Fig. 1c).

### Correlation between biglycan mRNA expression in adipose tissues and metabolic parameters, abdominal fat distribution, or adipocyte size

In the entire cohort (*n* = 91), biglycan mRNA level in adipose tissues (especially in VAT) showed strong positive correlations with adipocyte size and most of metabolic parameters we examined and a significant negative correlation with serum adiponectin concentration (Table 2). In addition, in both fat depots, biglycan mRNA expression significantly correlated with the TAT, VAT, and SAT areas assessed by abdominal CT; however, it did not correlate with VSR, an index of visceral obesity. Nevertheless, all the significant correlations between adipose tissue biglycan mRNA and metabolic parameters, adipocyte size, or abdominal fat distribution were not present after adjusting for BMI (data not shown).

### Biglycan mRNA expression correlates with the expression of genes related to inflammation and endoplasmic reticulum stress in human adipose tissues

In the entire study cohort (*n* = 91), we examined the relationship between adipose tissue biglycan mRNA levels and the expression of other genes (Table 3). Biglycan mRNA levels in adipose tissues, especially in VAT, showed a significant positive correlation with the expression of genes related to inflammation (cluster of differentiation 68 (CD68), TNF-α, tribbles homologs (TRIB)-1, and TRIB-2) and endoplasmic reticulum (ER) stress (C/EBP-homologous protein (CHOP) and TRIB-3); the statistical significance of the correlations mostly remained after adjustment for BMI.

### Induction of biglycan mRNA in human preadipocytes and differentiated adipocytes

Next, we tried to find an explanation for the elevated biglycan mRNA in obese adipose tissues via *in vitro* studies. Adipose tissue is composed of mature adipocytes (~50% of cells) and stromal cells including preadipocytes (~15% of cells), macrophages, immune cells, endothelial cell, and fibroblasts^21^. Although biglycan mRNA expression was much higher in non-adipocyte fraction than adipocytes (Fig. 1b), a major stromal cell producing biglycan is not known. We first suspected macrophage as a main biglycan-producing cell because macrophage infiltration increases in obese adipose tissues^22^. However, immunohistochemistry (IHC) on human adipose tissue samples revealed that cells detected with anti-CD68 (a marker for macrophage) antibody did not co-localize with cells expressing biglycan (Supplementary Figure 1), indicating that macrophages may not be the main contributor of increased biglycan mRNA in obese adipose tissues. Because it was not possible to obtain enough human adipose tissues to separately isolate and culture each type of stromal cells, we used human adipose tissue-derived stromal cells (mostly, if not all, preadipocytes) for this study.

**Figure 2:**
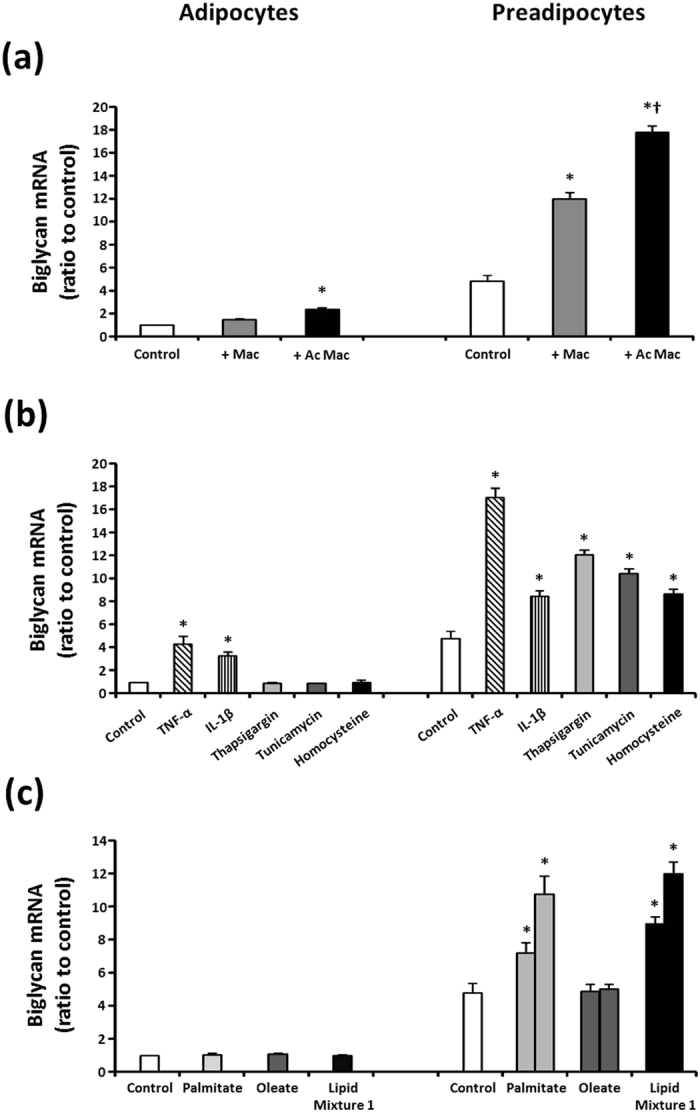
Induction of biglycan mRNA in human preadipocytes and differentiated adipocytes. (**a**) Induction of biglycan mRNA by co-culture with THP-1 macrophages. Human preadipocytes or differentiated adipocytes were co-cultured for 24 h with non-activated (Mac) or activated (AcMac) THP-1 macrophages or in medium alone (control). Cellular biglycan mRNA was measured using a real-time qPCR assay. Biglycan mRNA levels are normalized to 36B4 gene expression. Data are presented as the ratio to the biglycan mRNA level in the control adipocytes. Data are presented as the mean ± s.e.m. (*n* = 3). ^*^P < 0.05 vs control, ^†^*P* < 0.05 vs (+) Mac. Data were analyzed by ANOVA with Tukey’s *post hoc* test. (**b**) Biglycan mRNA induction by treating the cells with TNF-α (10 ng/ml), IL-1β (10 ng/ml), thapsigargin (500 nM), tunicamycin (2 μg/ml), or homocysteine (4 mM) for 24 h. (**c**) Biglycan mRNA induction by incubating the cells with palmitate (250 or 500 μM), oleate (250 or 500 μM), or lipid mixture 1 (added to the medium at concentrations of 5% or 10%) for 24 h.

In an effort to explain the elevated biglycan mRNA in obese adipose tissues, we incubated human preadipocytes as well as fully differentiated human adipocytes in a series of culture environments that could mimic the conditions during obesity (Fig. 2). Consistent with the results shown in Fig. 1b,c, prior to stimulation (control), the biglycan mRNA level was strikingly higher in preadipocytes than in adipocytes (Fig. 2a–c). Obesity is accompanied by infiltration of circulating monocytes into adipose tissue, resulting in accumulation of inflammed macrophages^22^. In adipose tissues of obese individuals, M2-type macrophages with remodeling capacity but extensively (even more than that in M1-type) secreting proinflammatory cytokines, have been identified^23^. Thus, to simulate obese human adipose tissues in this study, we utilized 1 μg/ml lipopolysaccharide (LPS) to generate activated THP-1 macrophages (AcMac), which expressed substantial quantity of proinflammatory cytokines TNF-α and IL-1β (data not shown). Compared with the control cells (cultured without macrophages), co-culture with non-activated THP-1 macrophages (Mac) significantly increased and AcMac even more increased biglycan mRNA in preadipocytes (Fig. 2a). In differentiated adipocytes, an elevation in biglycan mRNA expression was only significant following co-culture with AcMac. Moreover, incubation with the proinflammatory cytokines or ER stressors also markedly increased biglycan mRNA expression in preadipocytes. However, only TNF-α and IL-1β significantly increased biglycan mRNA in adipocytes; ER stressors failed to increase biglycan mRNA expression (Fig. 2b). Finally, incubation of preadipocytes with palmitate or lipid mixture 1, but not oleate, dose-dependently increased biglycan mRNA (Fig. 2c). In Mac and AcMac, these two lipids also significantly increased biglycan mRNA (data not shown). In contrast, incubation of adipocytes with the lipids failed to induce biglycan mRNA expression.

### Expression of biglycan mRNA in adipose tissues is not affected by hyperglycemia

**Figure 3:**
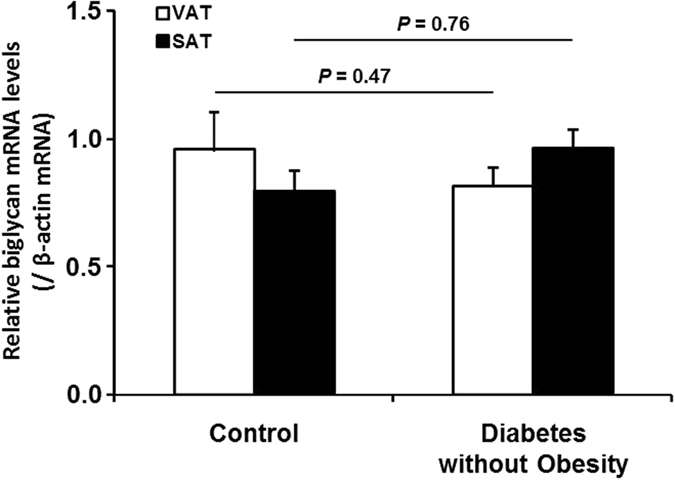
No difference in adipose biglycan mRNA expression between non-obese diabetic subjects and normal-weight controls. Biglycan mRNA levels in visceral (VAT) and subcutaneous (SAT) adipose tissue was measured in normal-weight women (*n* = 59; age 44.1 ± 8.2 years; BMI 22.5 ± 1.6 kg/m^2^) and non-obese type 2 diabetic women (*n* = 9; age 47 ± 8.7 years; BMI 25.7 ± 1.2 kg/m^2^). Biglycan mRNA levels were normalized to β-actin gene expression. Data were log-transformed before statistical analysis and are shown here as the mean ± s.e.m. on the original (back-transformed) scale.

We noted that adipose tissue biglycan mRNA was elevated in obese patients regardless of the presence of diabetes (Fig. 1a). Thus, we further examined the VAT and SAT biglycan mRNA in non-obese type 2 diabetic patients. In both fat depots of these patients, biglycan mRNA levels were not different from those of normal-weight control patients (Fig. 3). Moreover, incubation of human preadipocytes, differentiated adipocytes, and THP-1 macrophages in normal-glucose (5.5 mM) or high-glucose (25 mM) medium for 24 h did not affect the biglycan mRNA level (Supplementary Figure 2). It therefore appears that obesity, but not hyperglycemia, is a major factor in increasing biglycan mRNA in adipose tissues.

### Biglycan distribution in adipose tissues

**Figure 4:**
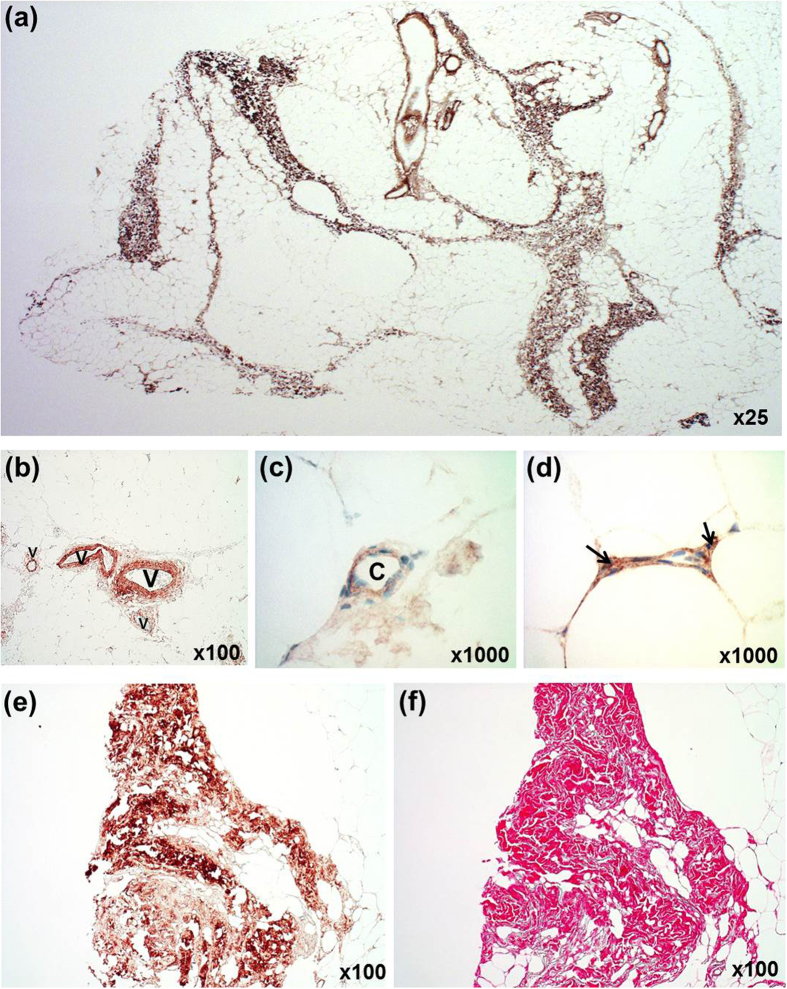
Biglycan staining in human adipose tissue. (**a**) Immunohistochemical localization of biglycan in adipose tissue. The tissue is counterstained with hematoxylin. (**b**,**c**) Biglycan immunoreactivity around venules (V) and a capillary (C). (**d**) Biglycan immunoreactivity around the margins of adipocytes and within the interior of non-adipocytes (arrows) residing within adipose tissues. (**e**,**f**) Colocalization of biglycan and collagen in a fibrotic area. Two serial sections of an adipose tissue sample were immunostained for biglycan (**e**) or stained for collagen with Sirius Red (**f**).

To determine the location of biglycan in adipose tissues, we performed IHC on VAT and SAT samples from obese women with or without type 2 diabetes and normal-weight women. Biglycan was abundantly expressed in adipose tissues with a similar staining pattern in VAT and SAT samples from the three groups. Consistent with the known interactions with ECM components such as collagens and elastins, intense biglycan staining was observed around blood vessels, in fibrotic areas, and within the septa of the adipose tissue itself (Fig. 4a). Especially at higher magnification, we observed biglycan accumulation in the connective tissues surrounding blood vessels (Fig. 4b) and in the cells lining the capillary wall (Fig. 4c). In addition, biglycan signal was detected in connective tissues surrounding some adipocyte borders (Fig. 4a,d) and within non-adipocytes (Fig. 4d) residing in adipose tissues. Biglycan also colocalized with collagen in fibrotic areas (Fig. 4e,f).

## Discussion

As adipose tissue grows, the ECM undergoes remodeling to accommodate the expansion. Thus, in obesity, adipose tissue ECM components undergo dynamic degradation and synthesis, with an overall increase in the expression of ECM components^24^. Although remodeling is necessary for adipose tissue expansion in obesity, increased ECM deposition may also cause adipose tissue dysfunction^25^. In the present study, we showed that the transcript level of biglycan was markedly higher in VAT and SAT of obese women than normal-weight women, which is consistent with a previous report of elevated biglycan mRNA expression in omental adipose tissue of obese human subjects^20^. Furthermore, we observed that adipose tissue biglycan mRNA expression was not increased by the presence of type 2 diabetes in either obese or normal-weight subjects. These findings indicate that, in human obesity, biglycan expression is primarily associated with adipose tissue expansion rather than with associated pathologies such as diabetes or hyperglycemia.

In contrast to *Psammomys obesus*, in which biglycan mRNA expression is higher in VAT than in SAT^16^, its expression was similar in the two fat depots in our study subjects (regardless of obesity or diabetes); this disparity is likely due to species differences. In both rodents^16^ and our human patients, biglycan was expressed in both adipocyte and SV cell fraction of adipose tissue, although it was more predominant in the latter. Accordingly, we detected biglycan immunoreactivity in the cells lining the capillary wall and in the non-adipocytes residing within adipose tissues. These findings indicate that SV cells, rather than mature adipocytes, contribute to excess biglycan production in the adipose tissues of obese subjects. The SV cell fraction of adipose tissue contains a variety of cells, including preadipocytes, endothelial cells, macrophages, and immune cells. At first, we expected that macrophages are the main contributor to the high expression of biglycan mRNA since macrophages are also increased in obese adipose tissues^22^. However, it is unlikely because biglycan and CD68 immunoreactivity did not colocalize in IHC of human adipose tissues. Separation of each cell type in the SV cell fraction would be helpful to determine the major cells and regulatory mechanisms responsible for elevated biglycan mRNA; however, it was limited to obtain human adipose tissues at quantities enough to perform this procedure. As an alternative, we observed that in human preadipocytes, stromal cells consisting 15–50% of cells in adipose tissue^21^, the biglycan mRNA level was more than five times higher than that in human differentiated adipocytes.

In addition to an intimate association with obesity, the biglycan mRNA level in each fat depot strongly and positively correlated with the expression of genes related to inflammation and ER stress, which are increased in obese adipose tissues. These results suggest that an altered local microenvironment may affect biglycan expression in obese adipose tissues. Indeed, co-culture with macrophages and addition of inflammatory cytokines, ER stressors, or free fatty acids to the culture medium markedly induced biglycan gene expression. Again, this phenomenon was more prominent in human preadipocytes than differentiated adipocytes, in accordance with the observation that the biglycan mRNA level was higher in SV cells than in the adipocyte fraction.

The expression level of biglycan mRNA in adipose tissues, especially in VAT, showed remarkably strong positive correlations with parameters related to insulin resistance as well as indices of adiposity such as BMI, abdominal fat areas, and adipocyte size in fat depots. It thus appears that the three factors—adipose tissue expansion, biglycan expression in adipose tissues, and insulin resistance—are closely inter-related, although the causality is obscure. Biglycan upregulation in adipose tissues may be involved in insulin resistance associated with obesity. Data from studies in rodents support this possibility: increased biglycan transcript levels in response to high-fat feeding in the adipose tissues of db/db mice^18^, decreased fasting insulin levels and HOMA-IR in biglycan knockout mice^19^, and improved glucose tolerance in biglycan knockout mice on high-fat diet relative to wild-type mice^20^.

The soluble form of biglycan acts as an endogenous ligand of TLR2/4 in macrophages^12^,^13^,^14^,^15^, leading to the release of proinflammatory cytokines, such as TNF-α and IL-1β^12^,^14^, and various chemoattractants for neutrophils and macrophages^14^,^15^. Furthermore, macrophages appear to induce biglycan synthesis in adipocytes and other cells residing within adipose tissues, as shown in the present study. Similar to the way that biglycan amplifies the inflammatory response in sterile inflammation, as in lupus nephritis^10^, biglycan may initiate and exacerbate the inflammatory reaction in adipose tissues of obese individuals, acting both in an autocrine and paracrine manner. In agreement with this, concomitant increases in biglycan and inflammatory gene expression are reported in the adipose tissues of obese patients^20^ and high-fat diet-fed mice^17^. In addition, high-fat diet-induced inflammatory gene expression is reduced in biglycan knockout mice^17^,^26^. However, in order to extend the current findings to this speculation, further studies including measurements of soluble biglycan levels in blood and culture media are required.

Despite marked upregulation of biglycan mRNA expression in the adipose tissues of obese patients, biglycan immunoreactivity was similar in the tissues of obese and normal-weight subjects. This may be because biglycan is synthesized as a precursor protein; to generate the mature form, an N-terminal pro-peptide is cleaved by proteases^27^. Secreted biglycan is sequestered through interactions of the core protein or glycosaminoglycan side chains with diverse components of the ECM^7^,^8^,^9^. Upon tissue stress or injury, biglycan is released from the ECM^11^ due to the activity of proteolytic enzymes^27^,^28^,^29^,^30^,^31^. Accordingly, in obesity, it is tempting to speculate that the accelerated proteolytic release of sequestered biglycan from the ECM in turn stimulates gene transcription to replace the depleted biglycan, thereby creating the discrepancy between biglycan protein and mRNA levels in adipose tissue. This possibility is supported by a study of white adipose tissue from db/db mice fed a high-fat diet that reported that matrix degradation genes and biglycan gene expression are upregulated in parallel^18^.

There are a few notable limitations to the present study. First, it is difficult to determine the causality of the observed relationships due to the cross-sectional study design. Second, our cohort was limited to female Koreans; therefore, our findings may not directly apply to other populations. Third, the numbers of subjects in our study groups were small and consequently the study was not powerful enough to discern confounding factors in our analysis. Fourth, we used HOMA-IR instead of glucose clamp, the gold standard for measuring whole-body insulin sensitivity. However, HOMA-IR measurements have been shown to correlate well with results obtained using the glucose clamp method^32^. Finally, our obese patients underwent low-calorie diet for 7–10 day prior to bariatric surgery; the preparation for surgery could have compounding effects on most of the measurements in these subjects. Nonetheless, an elevation of the biglycan mRNA level in adipose tissues observed in our obese patients might be higher, not lower, without the calorie restriction prior to surgery. Thus, our main findings at least, still remain undiminished with the consideration of this surgery preparation.

In summary, the present study shows that biglycan gene expression is elevated in the adipose tissues of obese patients, regardless of diabetic status. Biglycan mRNA is higher in non-adipocytes than adipocytes; biglycan mRNA level is increased only in the SV cell fraction, but not in adipocytes, of obese adipose tissues. The biglycan mRNA level is strongly associated with several parameters related to indices for body adiposity and insulin resistance; however, the association with the latter disappears after adjusting for BMI. In both VAT and SAT fat depots, biglycan gene expression closely correlates with the expression of genes related to inflammation and ER stress. Furthermore, co-culture with macrophages and addition of inflammatory cytokines, ER stressors, and free fatty acids increase biglycan gene expression in human differentiated adipocytes but more prominently in preadipocytes. Given that biglycan is released from the ECM under conditions of tissue stress and that unsequestered biglycan is an endogenous ligand for TLR2/4, the relevance of the current findings to the pathogenesis of obesity-related disorders such as insulin resistance warrant further studies.

## Methods

### Study subjects and adipose tissue sampling

The study cohort consisted of 21 obese (BMI ≥ 30 kg/m^2^) women without diabetes, 11 obese women with type 2 diabetes, and 59 normal-weight women (BMI 18.5–25.0 kg/m^2^) (91 women in total). All obese patients underwent laparoscopic Roux-en-Y gastric bypass surgery at the Obesity Center of Inha University Hospital (Incheon, Korea). Four days before surgery, patients were admitted and underwent routine physical examinations, systematic biochemical analyses after fasting, and abdominal CT. In addition, normal-weight women who underwent elective abdominal surgery for benign conditions were recruited as controls from the Gynecology Unit of Asan Medical Center (Seoul, Korea). Separately, nine non-obese type 2 diabetic women (BMI 23.6–27.0 kg/m^2^; age 34–59) were recruited for the assessment of biglycan mRNA in adipose tissues. Women with evidence of malignancy or severe hepatic or renal disease and those who were pregnant or lactating were excluded. All subjects provided written informed consent at enrollment. Some of the samples obtained from these patients were analyzed in previous studies^33^,^34^. The study protocol was approved by the Institutional Review Boards of Asan Medical Center and Inha University Hospital. All applicable institutional regulations regarding the ethical use of human volunteers were followed.

Anthropometric measurements and BP were obtained as previously described^33^. During surgery, 2–5 g samples of VAT and SAT were removed as previously described^33^.

### Measurements of metabolic variables

Patients discontinued all diabetes and hypertension medications for 3 days prior to blood sampling. After a 12 h fast, blood samples were obtained, and plasma and serum were immediately separated. Circulating concentrations of cholesterol, glucose, insulin, hs-CRP, leptin, and adiponectin were measured as previously described^33^,^34^. The HOMA-IR was calculated as previously described^32^.

### Estimation of abdominal fat distribution

Abdominal fat distribution was assessed using CT as previously described^33^. Total abdominal adipose tissue, VAT, and SAT were assessed using cross-sectional scans that were centered on the L4–L5 vertebral disc space. In addition, VSR, the VAT:SAT ratio, was calculated as an index of visceral obesity^35^,^36^.

### Adipocyte and SV cell fractionation

We examined biglycan mRNA expression in adipocyte and SV cell fractions of adipose tissue. A subgroup of our study subjects participated in this study: 9 normal-weight women, 6 non-diabetic obese women, and 5 obese women with type 2 diabetes. During surgery, 10 to 15 g of VAT was removed and adipocyte and SV cell fractions were isolated as described previously^33^.

### Real-time quantitative PCR

RNA isolation and real-time quantitative PCR (qPCR) were performed as described previously^33^; the primer sequences are shown in Supplementary Table 2. The mRNA expression levels of biglycan, sirtuin 1 (SIRT1), TNF-α, IL-1β, CD68, autophage protein 5 (Atg5), beclin1, transforming growth factor-β (TGF-β), TRIB-1, TRIB-2, TRIB-3, and CHOP were normalized to that of β-actin or 36B4.

### Isolation and differentiation of human preadipocytes

Discarded subcutaneous tissues were obtained from the transverse rectus abdominis musculocutaneous flap of nine separately recruited women (BMI = 23.4 ± 2.3 kg/m^2^; age = 43.1 ± 7.0 years) undergoing breast reconstruction surgery. For isolation of human preadipocytes, the SV cell fraction was re-suspended in erythrocyte lysis buffer (154 mM NH_4_Cl, 10 mM KHCO_3_; and 0.1 mM EDTA, pH 7.4) and centrifuged at 250 g for 10 min. After washing, the cells were suspended in DMEM supplemented with 10% fetal bovine serum (FBS) and used for cell culture at passage 2 to eliminate non-preadipocyte cell contamination. Preadipocytes were differentiated into adipocytes as previously described^34^.

To investigate the effects of proinflammatory cytokines and ER stressors on biglycan expression, human preadipocytes and fully differentiated adipocytes were incubated in serum-free medium for 24 h with TNF-α, IL-1β, tunicamycin, thapsigargin, or homocysteine. The effects of cell incubation with palmitate, oleate, or lipid mixture 1 (product number L0288; Sigma, St. Louis, MO; its lipid content consists of 2 μg/ml arachidonic acid, 10 μg/ml of each of linoleic, linolenic, myristic, oleic, palmitic, and stearic acid, and 0.22 mg/ml cholesterol) were also examined. Biglycan mRNA levels were measured in cell lysates with qPCR.

### Co-culture of human preadipocytes or differentiated adipocytes with macrophages

Human myelomonocytic (THP-1) cells were differentiated to macrophages via culturing with 10 ng/ml phorbol-12-myristate-13-acetate (Sigma) for 24 h and then cultured for an additional 24 h with or without 1 μg/ml LPS (Sigma) to prepare activated (AcMac) or non-activated (Mac) THP-1 macrophages, respectively. Co-culture was performed by utilizing transwell system, placing human preadipocytes or fully differentiated adipocytes in the lower well and THP-1 macrophages on the transwell inserts. Human preadipocytes or differentiated adipocytes were grown in 6-well dishes (the lower well); the Mac or AcMac was plated at 1 × 10^6^ cells on a polyester membrane insert (BD Falcon, New York, NC) with pore size 0.4 μm and pore density 4 × 10^6^ per cm^2^. Human preadipocytes or fully differentiated adipocytes were co-cultured with Mac or AcMac for 24 h in serum-free DMEM and then harvested to determine biglycan mRNA expression.

### Immunohistochemistry and Sirius Red staining

To examine biglycan distribution in adipose tissue, small pieces of VAT and SAT were fixed with 4% formalin and embedded in paraffin. The paraffin sections were subjected to automated IHC with the Benchmark XT slide staining system together with the OptiView DAB IHC Detection Kit (Ventana Medical Systems, Inc., Tucson, AZ) according to the manufacturer’s protocol. The sections incubated with anti-biglycan antibody (Abcam, Cambridge, UK) or anti-CD68 antibody (Dako, Glostrup, Denmark) were exposed to 3,3′-diaminobenzidine (DAB) and the nuclei were counterstained with hematoxylin. To examine colocalization, sequential serial sections were subjected to IHC staining for biglycan, CD68, or Sirius Red (Polyscience, Inc., Warrington, PA) collagen stain as previously described^37^. Tissues were examined and photographed with the Carl Zeiss Axio Imager A1 microscope outfitted with the Carl Zeiss Axio Cam ICc3 (Carl Zeiss, Göttingen, Germany).

### Adipocyte size measurement

We estimated adipocyte size from the mean cross-sectional area of adipocytes in hematoxylin and eosin-stained sections of adipose tissues. Paired samples of VAT and SAT were obtained from a subgroup of subjects. On each hematoxylin and eosin slide, digital photomicrographs (×100) were taken in two separate areas (containing a total of 500–1,000 adipocytes). The cross-sectional areas of the adipocytes were estimated using Image Pro Plus software (Media Cybernetics, version 4.5.0.29) in an average of two separate areas from each slide.

### Statistical analyses

A Student’s paired or unpaired *t*-test was performed as indicated for pairwise group comparisons, and one-way ANOVA followed by Tukey’s *post hoc* test for significance was used for multiple comparisons. Data are presented as mean ± s.d. for normally distributed parameters such as age, BMI, and adipocyte size. Non-normally distributed data, such as the HOMA-IR index, mRNA levels of biglycan, SIRT1, TNF-α, IL-1β, CD68, Atg5, beclin1, TGF-β, TRIB-1–3, and CHOP in adipose tissues, and circulating levels of glucose, insulin, triglyceride, hs-CRP, leptin, and adiponectin, were log-transformed prior to analysis to generate a normal distribution. These data are presented as the mean ± s.e.m. on their original untransformed scales. Correlation coefficients between measures were calculated using Pearson’s correlation. For all tests, *P* < 0.05 was considered to be statistically significant. All statistical analyses were performed using SPSS (version 19.0; IBM, Chicago, IL).

## Additional Information

**How to cite this article**: Kim, J. *et al*. Enhanced biglycan gene expression in the adipose tissues of obese women and its association with obesity-related genes and metabolic parameters. *Sci. Rep.* **6**, 30609; doi: 10.1038/srep30609 (2016).

## Supplementary Material

Supplementary Information

## Figures and Tables

**Table 1:** Metabolic parameters, abdominal fat distribution, and adipocyte size of the non-diabetic obesity, obesity with diabetes, and normal-weight control groups.

	Control	Obesity	Obesity with Diabetes
*n*	59	21	11
Age (yr)^a^	44.1 ± 8.2	32.1 ± 8.9^*^	45.8 ± 8.6^†^
BMI (kg/m^2^)^a^	22.5 ± 1.6	40.2 ± 5.1^*^	33.8 ± 4.7^*,†^
Systolic BP (mmHg)^a^	119.1 ± 16.1	139.7 ± 15.0^*^	137.6 ± 16.7^*^
Diastolic BP (mmHg)^a^	72.5 ± 11.7	83.5 ± 9.2^*^	85.0 ± 8.5^*^
Glucose (mg/dl)^b^	105.3 ± 3.1	96.1 ± 3.0	204.7 ± 15.0^*,†^
Insulin (μU/ml)^b^	4.8 ± 0.5	24.3 ± 2.5^*^	22.7 ± 4.7^*^
HOMA-IR^b^	1.92 ± 0.51	5.84 ± 0.64^*^	12.1 ± 3.0^*^
Total cholesterol (mg/dl)^a^	164.9 ± 33.5	183.2 ± 33.1	194.7 ± 53.5^*^
HDL cholesterol (mg/dl)^a^	40.9 ± 11.8	48. 6 ± 9.0^*^	38.8 ± 8.7^†^
LDL cholesterol (mg/dl)^a^	91.7 ± 28.6	116.2 ± 26.6^*^	124.5 ± 37.3^*^
Triglyceride (mg/dl)^b^	77.4 ± 6.3	130.7 ± 13.8^*^	290.9 ± 87.5^*,†^
hs-CRP (mg/dl)^b^	0.08 ± 0.02	0.58 ± 0.11^*^	0.65 ± 0.22^*^
Leptin (ng/ml)^b^	5.4 ± 0.5	39.6 ± 4.1^*^	23.6 ± 5.0^*,†^
Adiponectin (μg/ml)^b^	6.84 ± 0.66	3.04 ± 0.45^*^	5.02 ± 1.56
HbA1c (%)^a^	N/A	5.80 ± 0.53	9.44 ± 2.05^†^
*Adipocyte size*^*c*^			
VAT adipocyte (μm^2^)^a^	3821 ± 1752	7644 ± 4060^*^	7052 ± 2958^*^
SAT adipocyte (μm^2^)^a^	5716 ± 2056^#^	8454 ± 3090^*^	6726 ± 2790
*Abdominal CT*			
TAT area (cm^2^)^a^	346.7 ± 179.7	684.3 ± 157.4^*^	566.3 ± 163.3^*^
VAT area (cm^2^)^a^	83.7 ± 45.7	143.1 ± 56.3^*^	217.7 ± 56.6^*,†^
SAT area (cm^2^)^a^	213.9 ± 85.7	540.1 ± 155.9^*^	348.6 ± 127.3^*,†^
VSR^a^	0.39 ± 0.14	0.30 ± 0.18	0.66 ± 0.20^*,†^

^a^Data are shown as the mean ± s.d. ^b^Data were log-transformed for statistical analysis. Data are shown as the mean ± s.e.m. on the original (back-transformed) scale. ^c^Adipocyte sizes in VAT and SAT were measured in normal-weight control (*n* = 25), non-diabetic obese (*n* = 20), and diabetic obese women (*n* = 10). ^*^P < 0.05 vs control, ^†^*P* < 0.05 vs obesity group by ANOVA with Tukey’s post hoc test, ^#^*P* *<* 0.05 vs VAT of the same group by paired t-test. BP, blood pressure; HOMA-IR, homeostasis model assessment of insulin resistance; hs-CRP, high-sensitive C-reactive protein; CT, computerized tomography; TAT, total adipose tissue; VAT, visceral adipose tissue; SAT, subcutaneous adipose tissue; VSR, the ratio of the visceral adipose tissue area to the subcutaneous adipose tissue area; N/A, not available.

**Table 2:** Correlation of biglycan mRNA expression in adipose tissues with metabolic parameters, abdominal fat distribution, and adipocyte size in study participants.

	VAT		SAT	
	*r*	*p*	*r*	*p*
BMI	0.571	<0.001	0.508	<0.001
Systolic BP	0.335	0.001	0.292	0.005
Diastolic BP	0.245	0.020	0.205	0.052
Glucose	0.219	0.037	0.181	0.086
Insulin	0.380	<0.001	0.328	0.002
HOMA-IR	0.386	<0.001	0.330	0.002
Total cholesterol	0.244	0.020	0.143	0.176
HDL cholesterol	0.118	0.283	0.184	0.093
LDL cholesterol	0.369	0.001	0.371	<0.001
Triglyceride	0.344	0.004	0.290	0.016
hs-CRP	0.495	<0.001	0.410	<0.001
Leptin	0.495	<0.001	0.444	<0.001
Adiponectin	−0.339	0.002	−0.252	0.021
Adipocyte size^*^	0.310	0.021	0.331	0.014
*Abdominal CT*				
TAT area	0.540	<0.001	0.343	0.015
VAT area	0.462	0.001	0.330	0.019
SAT area	0.507	<0.001	0.368	0.009
VSR	0.085	0.557	0.097	0.501

*n* = 91, *r* = Pearson correlation coefficient. ^*^n = 55. BP, blood pressure; HOMA-IR, homeostasis model assessment of insulin resistance; hs-CRP, high-sensitive C-reactive protein; CT, computerized tomography; TAT, total adipose tissue; VAT, visceral adipose tissue; SAT, subcutaneous adipose tissue; VSR, the ratio of the visceral adipose tissue area to the subcutaneous adipose tissue area.

**Table 3:** Correlation between the expression of biglycan mRNA and other genes in adipose tissues of study participants.

	VAT		SAT	
	*r*	*r*^*#*^	*r*	*r*^*#*^
SIRT1	0.038	0.031	−0.060	−0.014
TNF-α	0.516^*^	0.413^*^	0.100	−0.026
IL-1β	0.171	0.070	−0.100	−0.065
CD68	0.581^*^	0.434^*^	0.480^*^	0.380^*^
Atg5	0.080	−0.043	0.199	0.288^*^
Beclin1	0.192	0.089	0.219^*^	0.078
TGF-β	0.246^*^	0.124	0.179	−0.115
TRIB-1	0.257^*^	0.328^*^	0.175	0.006
TRIB-2	0.354^*^	0.367^*^	0.174	0.263
TRIB-3	0.384^*^	0.420^*^	0.368^*^	0.282^*^
CHOP	0.502^*^	0.437^*^	0.419^*^	0.220

*n* = 91, ^*^P < 0.05. *r* = Pearson correlation coefficient. *r*^#^ = Pearson correlation coefficient after adjustment for BMI. VAT, visceral adipose tissue; SAT, subcutaneous adipose tissue; SIRT1, sirtuin 1; TNF-α, tumor necrosis factor alpha; IL-1β, interleukin 1 beta; CD68, cluster of differentiation 68; Atg5, autophage protein 5; TGF-β, transforming growth factor β; TRIB, tribbles homolog; CHOP, C/EBP-homologous protein.
