# Supplementary material for: Enhanced biglycan gene expression in the adipose tissues of obese women and its association with obesity-related genes and metabolic parameters
Source: Sci Rep. 2016 Jul 28;6:30609. doi: 10.1038/srep30609 (PMC4964581; doi:10.1038/srep30609)
Supplement: Supplementary Information [file srep30609-s1.pdf]

Supplementary Information

Enhanced biglycan gene expression in the adipose tissues of obese women and its association with obesity-related genes and metabolic parameters

Jimin Kim[1], Seul Ki Lee[1], Ji-min Shin[1], Un-woo Jeoun[1], Yeon Jin Jang[1*], Hye Soon Park[2], Jong-Hyeok Kim[3], Gyung-Yub Gong[4], Taik Jong Lee[5], Joon Pio Hong[5], Yeon Ji Lee[6] and Yoon-Suk Heo[7]

[1]Department of Physiology, Cell Dysfunction Research Center, University of Ulsan College of Medicine, Seoul, Korea; [2]Department of Family Medicine, University of Ulsan College of Medicine, Seoul, Korea; [3]Department of Obstetrics and Gynecology, University of Ulsan College of Medicine, Seoul, Korea; [4]Department of Pathology, University of Ulsan College of Medicine, Seoul, Korea; [5]Department of Plastic Surgery, University of Ulsan College of Medicine, Seoul, Korea; [6]Department of Family Medicine, Inha University, College of Medicine, Incheon, Korea; [7]Department of General Surgery, Inha University, College of Medicine, Incheon, Korea

	Control	Obesity	Obesity with Diabetes
n	59	21	11
Menopause	8	1	4
Gynecological surgery patients			
Myoma	25		
Leiomyoma	14		
Teratoma	2		
Cystadenoma	1		
Others	17		
Duration of diabetes			
2−9 yr			3
≥10 yr			8
Receiving treatment			
Hypertension	0	0	7
Beta-receptor blocker			1
Angiotensin receptor blocker			5
Calcium channel blocker			1
Diuretics			1
Diabetes	0	0	11
Metformin			7
Sulfonamides			3
Glucosidase inhibitor			2
Dyslipidemia	0	0	3
Statins	0	0	3

Table S1. Clinical characteristics of the non-diabetic obesity, obesity with type 2 diabetes, and normal-weight control groups.

Gene	Forward primer (5'-3')	Reverse primer (5'-3')
Biglycan	GGTGGCTAGGTCTCCCCTTA	CACGTTGCACGGTGTTTCTT
SIRT1	CAGTGTCATGGTTCCTTTGC	CCTAGGACATCGAGGAACTACC
TNF-α	TCTCATGCACCACCATCAAGGACT	TGACCACTCTCCCTTTGCAGAACT
IL-1β	ACAGCTGGAGAGTGTAGATCC	CTTGAGAGGTGCTGATGTACC
CD68	CTACATGGCGGTGGAGTACAA	ATGATGAGAGGCAGCAAGATGG
Atg5	TGCTTCGAGATGTGTGGTTTGGACG	ATAATGCCATTTCAGTGGTGTGCCT
Beclin1	GGCTGAGAGACTGGATCAGG	CTGCGTCTGGGCATAACG
TGF-β	CCCAGCATCTGCAAAGCTC	GTCAATGTACAGCTGCCGCA
TRIB1	GCTGCAAGGTGTTTCCCATTA	CTCTTCCGGCTTCGCACATA
TRIB2	TTGAGCGAAGCTATGGGGAC	TGACCCGAGTCCTCTCTTCG
TRIB3	GACCGTGAGAGGAAGAAGCTGG	TGCCTTGCCCGAGTATGAGG
CHOP	AGGGAGAACCAGGAAACGGAAACA	CTGACTGGAATCTGGAGAG
β-actin	GACGGGGTCACCCACAC	GTGGTGGTGAAGCTGTAGCC
36B4	CCTGAGTGATCTGCAGCTG	CACCTGCTGGATGACCAGC

Table S2. Primer sequences used for real-time qPCR analyses

SIRT1, sirtuin 1; TNF-α, tumor necrosis factor alpha; IL-1β, interleukin 1 beta; CD68, cluster of differentiation 68; Atg5, autophage protein 5; TGF-β, transforming growth factor β; TRIB, tribbles homolog; CHOP, C/EBP-homologous protein.

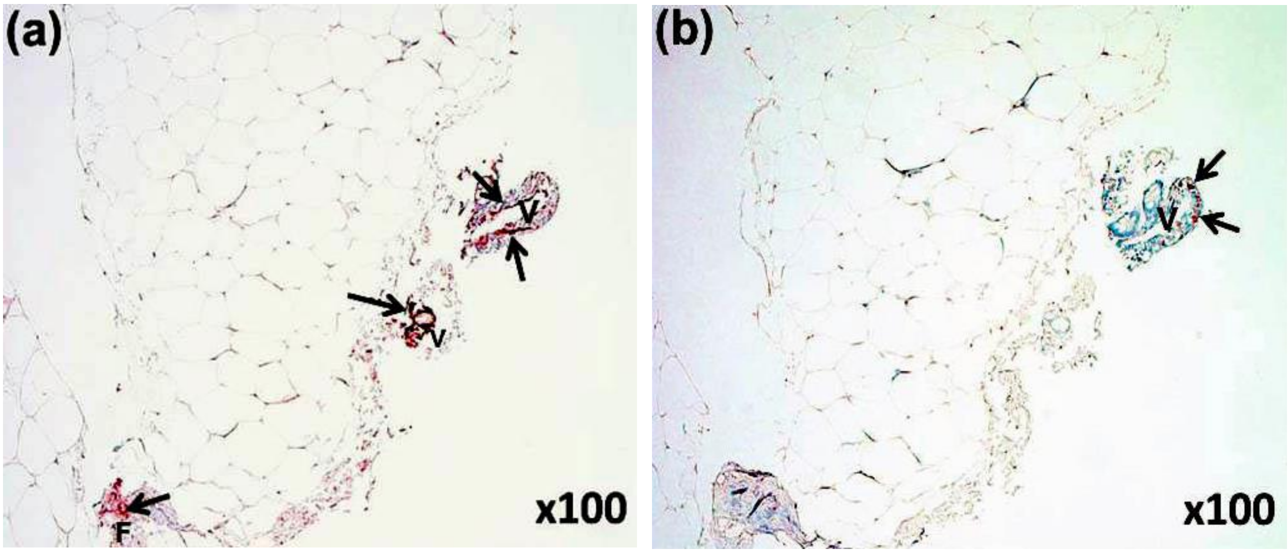

Figure S1. No colocalization between biglycan and CD68 in human adipose tissues. Two serial sections of an adipose tissue sample were immunostained for biglycan (**a**) or CD68 (**b**). Biglycan immunoreactivity around venules (V), fibrotic area (F), and around the margins of adipocytes (**a**). CD68 immunoreactivity around only outer venules (V) and interior non-adipocytes residing within adipose tissues (**b**). Positive detection area of biglycan and CD68 were presented by arrows.

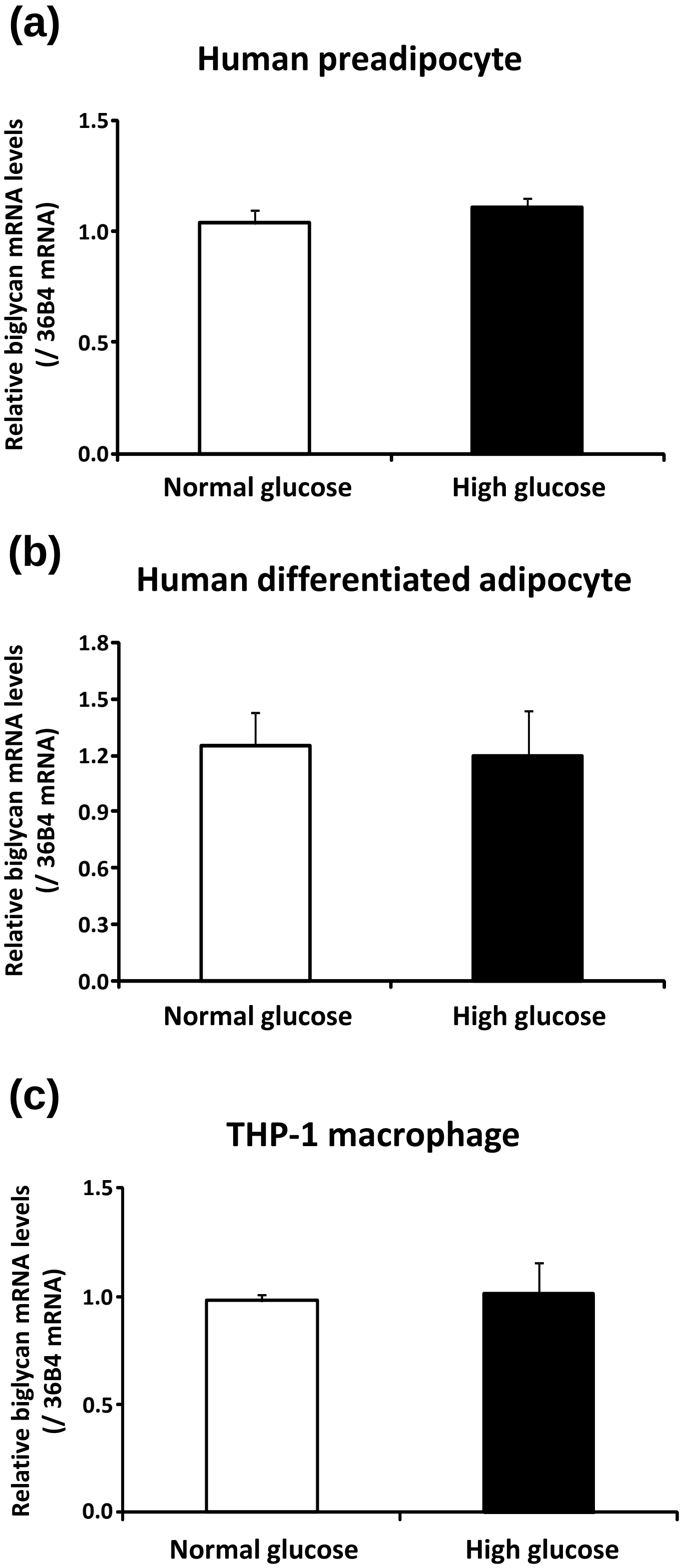

Figure S2. No effect of glucose level in the culture medium on biglycan mRNA expression. Human preadipocytes (**a**), differentiated adipocytes (**b**), and THP-1 macrophages (**c**) were incubated for 24 h in serum-free media with a normal (5.5 mM) or high (24 mM) glucose concentration. Cellular biglycan mRNA was measured using a real-time qPCR assay. Biglycan mRNA levels were normalized to 36B4 gene expression. Data are presented as the mean ± s.e.m. (n = 3).
